# Higher Prevalence of Subclinical Hypothyroidism in Croatian War Veterans: A Cross-Sectional Study

**DOI:** 10.3390/epidemiologia7040113

**Published:** 2026-08-18

**Authors:** Dino Pavičić, Zrinka Čolak Romić, Josip Čurić, Ivija Rajković, Irijana Rajković, Anton Glasnović, Ingrid Prkačin, Dario Rahelić, Tomislav Bulum, Sanda Tešanović, Branimir Kutuzović Hackenberger

**Affiliations:** 1 Polyclinic Medikol, Voćarska Cesta 106, 10000 Zagreb, Croatia; dino.pavicic@medikol.hr (D.P.);; 2 Department of Neurology, Clinical Hospital Dubrava, Avenija Gojka Šuška 6, 10000 Zagreb, Croatia; 3 Croatian Institute for Brain Research, School of Medicine, University of Zagreb, Šalata 12, 10000 Zagreb, Croatia; 4 Vuk Vrhovac University Clinic for Diabetes, Endocrinology and Metabolic Diseases, Merkur University Hospital, Dugi dol 4a, 10000 Zagreb, Croatia; 5 School of Medicine, Catholic University of Croatia, Ilica 242, 10000 Zagreb, Croatia; 6 School of Medicine, Josip Juraj Strossmayer University, Josipa Huttlera 4, 31000 Osijek, Croatia; 7 School of Medicine, University of Zagreb, Šalata 3, 10000 Zagreb, Croatia; 8 Polyclinic Hygea, Pera Čingrije 2, 20000 Dubrovnik, Croatia; 9 Department of Biology, University of Osijek, Cara Hadrijana 8A, 31000 Osijek, Croatia

**Keywords:** war veterans, subclinical hypothyroidism, TSH, thyroid function, cross-sectional study

## Abstract

Background/Objectives: Long-term thyroid function in war veterans remains insufficiently characterized. This study examined the prevalence of thyroid dysfunction in Croatian Homeland War veterans compared to controls. Methods: Cross-sectional study comparing 337 male veterans (mean age 62.4 years) with 335 age-comparable male controls (mean age 61.7 years). Thyroid function was assessed via TSH and FT4. The main outcome of interest was the prevalence of subclinical hypothyroidism (TSH over 4.0 mIU/L with normal FT4). Results: Subclinical hypothyroidism was more prevalent in veterans (12.5%, 42/335) than controls (5.2%, 16/305; OR = 2.59, 95% CI: 1.42–4.71, p = 0.001). After adjustment for age and BMI, the association remained significant (adjusted OR = 2.25, 95% CI: 1.19–4.23, p = 0.012). Mean TSH was modestly higher in veterans (2.53 ± 1.47 vs. 2.06 ± 1.10 mIU/L, *p* = 0.001), with both groups’ mean values within the normal reference range. Other biochemical parameters showed no significant differences. Conclusions: Veterans showed a higher prevalence of subclinical hypothyroidism compared to controls. The clinical significance of this finding requires further investigation, as mean TSH values remained within normal limits in both groups. The cross-sectional design precludes causal inference, and unmeasured confounders may contribute to the observed association.

## 1. Introduction

Combat exposure is associated with long-term health consequences, including elevated rates of post-traumatic stress disorder (PTSD), cardiovascular disease, and various somatic complaints. The hypothalamic–pituitary–thyroid (HPT) axis responds to both acute and chronic stress, though the direction and magnitude of stress-induced thyroid alterations remain inconsistent across studies.

Thyroid dysfunction represents an increasingly recognized health concern among war veterans, potentially reflecting the complex interaction between environmental exposures, chronic stress, and comorbid conditions. Both hypothyroidism and hyperthyroidism have been reported at higher rates in veteran populations compared to the general population, although findings remain heterogeneous across studies [1,2]. Chronic psychological stress, including PTSD, may influence the HPT axis, leading to alterations in thyroid hormone levels and autoimmune activity [3]. Additionally, exposure to environmental toxins, such as burn pit emissions, heavy metals, and chemical agents, has been suggested as a contributing factor to thyroid dysfunction [2].

Autoimmune thyroid diseases, particularly Hashimoto’s thyroiditis and Graves’ disease, may be more prevalent in this population, possibly due to immune dysregulation triggered by stress and environmental insults [4]. Furthermore, metabolic comorbidities frequently observed in veterans, including obesity and type 2 diabetes, may further complicate thyroid function and its clinical manifestations. Early recognition and appropriate screening for thyroid disorders in veterans are essential, especially in those presenting with nonspecific symptoms such as fatigue, weight changes, or mood disturbances.

The Croatian Homeland War (1991–1995) involved approximately 500,000 combatants, many of whom experienced significant trauma. While psychiatric outcomes in this population have been documented, systematic investigation of thyroid function has been limited. This study aimed to compare thyroid function parameters in Croatian war veterans with age-comparable controls. The main objective was to determine the prevalence of subclinical hypothyroidism in both groups. Additional objectives included examining continuous TSH and FT4 differences and exploring potential confounders.

## 2. Methods

### 2.1. Study Design and Participants

This cross-sectional study included 672 male participants: 337 Croatian Homeland War veterans and 335 controls. Veterans were recruited from veteran associations and healthcare facilities. Controls were recruited from primary healthcare centers. Inclusion criteria included male sex, age between 50 and 75 years, and ability to provide informed consent. Exclusion criteria included current thyroid medication and known thyroid malignancy. Veterans and controls were not individually paired; both groups were recruited within the same eligibility range (male sex, age 50–75 years), representing group-level (frequency) rather than individual case–control matching. As a small residual age difference remained between groups (Table 1), age was additionally included as a covariate in the adjusted logistic regression analysis. Veterans with PTSD and other mental illnesses were included in the study.

### 2.2. Data Collection

Demographic and clinical data (age, height, weight, smoking status) were collected using a self-administered questionnaire completed by participants as part of a routine health screening visit. Blood samples were collected after overnight fasting for biochemical parameters: TSH (reference: 0.4–4.0 mIU/L), FT4, total cholesterol, HDL cholesterol, LDL cholesterol, triglycerides, aspartate aminotransferase (AST), alanine aminotransferase (ALT), gamma-glutamyl transferase (GGT), fasting glucose, and uric acid. T4 was measured using Pure Analyzer Cobas (Roche) with laboratory reference range 60–120 [units]. This reference range differs from typical immunoassay ranges (10–25 pmol/L), likely reflecting assay methodology differences. For between-group comparisons, the consistent methodology ensures the validity of relative differences. Those with TSH over 4.0 mIU/L with FT4 within the reference range were classified as subclinical hypothyroidism; those with TSH over 10.0 mIU/L with low FT4 were classified as overt hypothyroidism, while those with TSH below 4 mIU/L had suppressed TSH.

### 2.3. Statistical Analysis

Analyses were performed using R version 4.3.0. The main outcome of interest was the prevalence of subclinical hypothyroidism. Between-group comparison used Fisher’s exact test. Odds ratios (OR) with 95% confidence intervals were calculated. Logistic regression was adjusted for age and BMI to account for the residual between-group age difference (Table 1), despite groups being recruited from the same eligibility range rather than individually paired. In additional analyses, continuous variables were compared using Welch’s *t*-test; given the large sample size in each group (*n* > 300), parametric testing is robust to the observed non-normality of TSH values via the Central Limit Theorem, and mean ± SD was retained for comparability with published population reference values, which are similarly reported as means. Effect sizes were reported as Cohen’s d, interpreted as: negligible (|d|<0.2), small (0.2≤|d|<0.5), medium (0.5≤|d|<0.8), and large (|d|≥0.8). Bayesian *t*-tests computed Bayes factors (BF_10_) as a sensitivity analysis, interpreted per anecdotal (1–3), moderate (3–10), strong (10–30), very strong (30–100), extreme (100). Given 11 biochemical comparisons, the Bonferroni-corrected significance threshold was α = 0.0045.

## 3. Results

### 3.1. Participant Characteristics

Groups were comparable in age, height, weight, and BMI (Table 1), and complete data were available for these variables in all participants; age and BMI were therefore included as covariates in the adjusted logistic regression model. Smoking status, in contrast, was available for only 254/672 participants (38%), which precluded its inclusion as a covariate in the adjusted model.

### 3.2. Prevalence of Subclinical Hypothyroidism

Table 2 shows the distribution of thyroid status by group. Subclinical hypothyroidism (TSH 4.0–10 mIU/L) was significantly more prevalent in veterans (12.2%) than in controls (5.2%).

The crude odds ratio for subclinical hypothyroidism in veterans vs. controls was 2.59 (95% CI: 1.42–4.71, p = 0.001). After adjustment for age and BMI, the association remained significant (2.25 (95% CI: 1.19–4.23, *p* = 0.01)).

### 3.3. Continuous Thyroid Parameters

Table 3 presents continuous TSH and FT4 values. Veterans showed modestly higher mean TSH (difference: 0.47 mIU/L), though both groups’ means were within the normal reference range (0.4–4.0 mIU/L).

**Figure 1:**
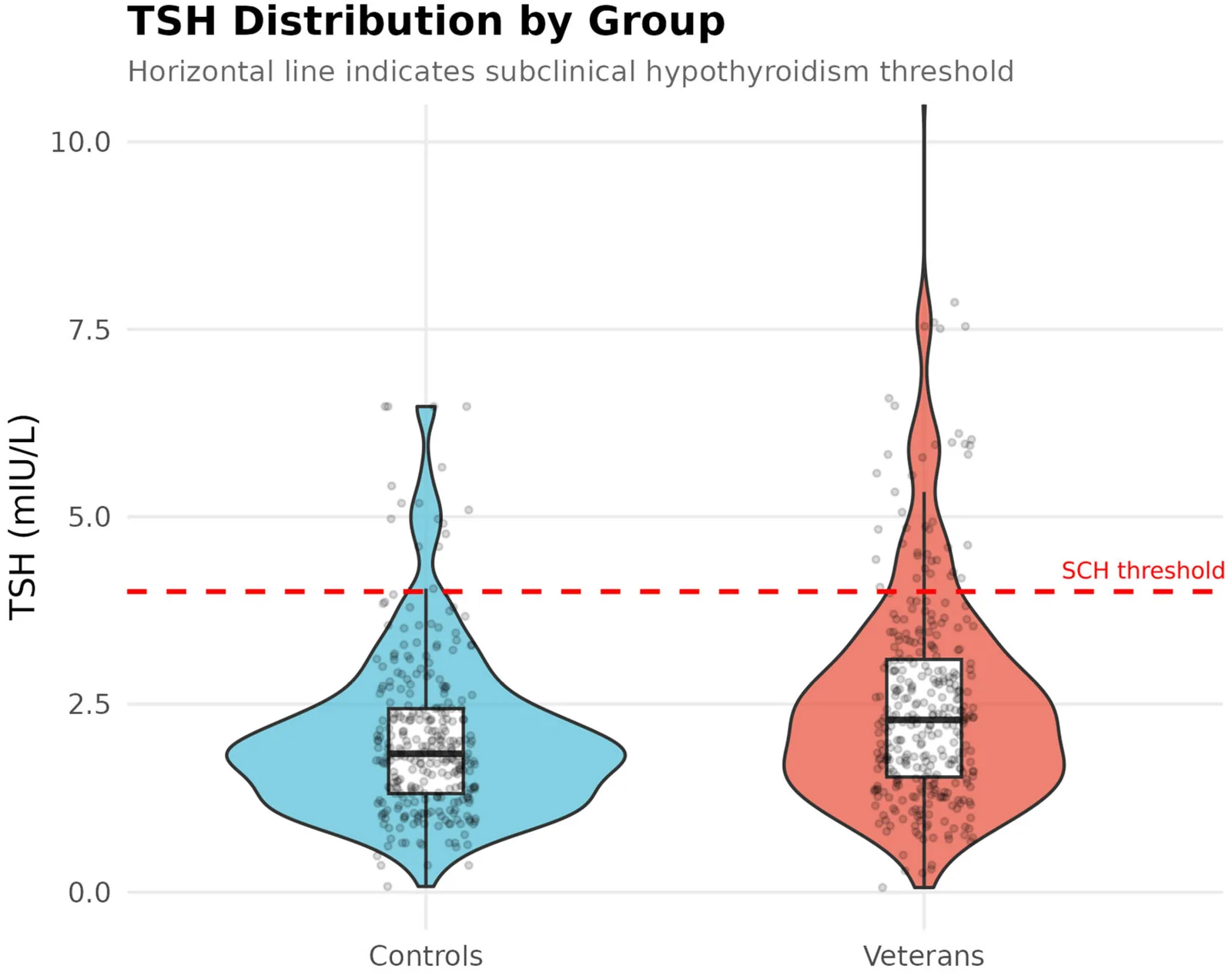
TSH distribution by group. Violin plots show the probability density of TSH values, with embedded boxplots indicating median and interquartile range. Individual data points are overlaid. The horizontal dashed red line indicates the subclinical hypothyroidism threshold (TSH 4.0 mIU/L). Veterans show a rightward shift in the distribution, with more individuals exceeding the threshold.

Figure 1 visualizes the TSH distribution in both groups, showing the rightward shift in the veteran distribution and the greater proportion of veterans exceeding the subclinical hypothyroidism threshold.

### 3.4. Other Biochemical Parameters

Other biochemical parameters showed no significant between-group differences after Bonferroni correction (Supplementary Table S1). Bayesian analysis provided evidence for the null hypothesis for most parameters (BF_10_ 0.33). 

## 4. Discussion

This cross-sectional study found that Croatian Homeland War veterans had approximately 2.5-fold higher odds of subclinical hypothyroidism compared to age-comparable male controls (12.5% vs. 5.2%; OR = 2.59, 95% CI: 1.42–4.71). This association persisted after adjustment for age and BMI (adjusted OR = 2.25, 95% CI: 1.19–4.23). Mean TSH was modestly but significantly higher in veterans (2.53 vs. 2.06 mIU/L, Cohen’s d = 0.36), though importantly, mean values in both groups remained within the normal reference range (0.4–4.0 mIU/L).

Sensitivity analyses using different TSH cutoffs showed consistent findings for thresholds up to 4.5 mIU/L. At higher cutoffs (TSH 5.0 mIU/L), point estimates remained elevated (OR = 2.20), but confidence intervals crossed unity, likely due to reduced statistical power from smaller sample sizes (Supplementary Table S2). Age-stratified analysis revealed notable heterogeneity in the veteran-control difference (Supplementary Table S3). The odds ratio for subclinical hypothyroidism was markedly higher in veterans aged 60–65 (OR = 11.57, 95% CI: 2.57–52.02, *p* = 0.0001) and those over 65 (OR = 11.00, 95% CI: 1.43–84.50, *p* = 0.005) compared to younger age groups, where no significant differences were observed. However, although the observed differences in TSH concentrations reached statistical significance, their absolute magnitude was relatively small and may not necessarily be clinically meaningful. Therefore, the findings should be interpreted with caution, as statistical significance does not automatically imply clinical significance, particularly when differences remain within or close to the established reference range.

The difference between groups was more pronounced in the upper portion of the TSH distribution: at the 90th percentile, veterans had TSH values of 4.42 mIU/L compared to 3.34 mIU/L in controls (difference: 1.08 mIU/L), and at the 95th percentile, the difference widened to 1.61 mIU/L (5.64 vs. 4.03 mIU/L). This suggests that the veteran-control difference is driven primarily by a subgroup with elevated TSH rather than a uniform shift in the entire distribution (Supplementary Table S4). In addition, TSH distributions were significantly non-normal in both groups (Shapiro–Wilk p 0.001; Supplementary Table S5).

Research indicates that the prevalence of subclinical hypothyroidism, defined by elevated serum TSH, in veterans may exceed that of the general population, primarily due to a combination of advancing age, high comorbidity burden, and specific service-related environmental exposures [5,6]. Our control group’s TSH (2.06 mIU/L) and subclinical hypothyroidism prevalence (5.2%) are consistent with published population norms according to the NHANES (National Health and Nutrition Examination Survey) US population data. Those data show the veteran group values above typical population ranges [5]. In large-scale longitudinal studies of veterans, approximately 7% to 10% of veterans exhibit elevated TSH levels, with significantly higher rates noted in female veterans and those over the age of 65 [7]. The clinical implications of these findings are multifaceted. Unlike the general population, where subclinical hypothyroidism is often asymptomatic, veterans frequently present with overlapping symptoms from conditions such as PTSD and traumatic brain injury (TBI), which can mask or mimic the neurocognitive symptoms of hypothyroidism [8].

Furthermore, recent evidence suggests a bidirectional link between chronic stress and thyroid dysfunction. Chronic stress has been shown to influence the HPT axis through complex neuroendocrine mechanisms, primarily mediated by activation of the hypothalamic–pituitary–adrenal (HPA) axis and increased cortisol secretion [3]. Elevated cortisol levels can suppress hypothalamic thyrotropin-releasing hormone (TRH) and pituitary TSH secretion, as well as impair peripheral conversion of thyroxine (T4) to triiodothyronine (T3), favoring the production of inactive reverse T3 [9]. These alterations may contribute to subtle thyroid dysfunction, including subclinical hypothyroidism. Additionally, chronic stress has been implicated in immune dysregulation, potentially increasing susceptibility to autoimmune thyroid diseases, which represent the most common underlying cause of subclinical hypothyroidism [4,10]. Although the causal relationship remains incompletely understood, accumulating evidence suggests that prolonged stress exposure may play a role in the development or exacerbation of subclinical thyroid dysfunction, particularly in vulnerable populations. TSH levels may also increase during depressive episodes, which are common in veterans, and high TSH levels complicate the treatment of depression [11]. Also, exposure to endocrine-disrupting chemicals, such as Agent Orange or per- and polyfluoroalkyl substances (PFAS) found on military installations, has been associated with alterations in thyroid function and may contribute to dysregulation of the HPT axis in military personnel [6].

Croatian war veterans were found to be more likely to develop hypertension than individuals in the general population, as well as diabetes, hyperlipidemia, coronary heart disease, malignancy, psychiatric diseases, and alcohol consumption. However, a significant difference was found in favor of the general population for hypothyroidism [12]. Inconsistent data regarding the relationship between thyroid status in veterans might be due to the multifaceted impact of stress on thyroid activity. This relationship is shaped by several variables, including the category and duration of the stressor, its severity and predictability, as well as the specific biological sample and the timing of hormonal assessment [13,14]. Studies investigating the human stress response have shown conflicting effects on peripheral thyroid hormone concentrations. Outcomes vary from reduced levels to elevated or even stable levels [15]. Such contradictions are also evident in laboratory-based stress experiments. The intricate relationship between stress and thyroid activity appears to be contingent upon the specific type and duration of the stressor. This complexity explains why the exact mechanisms governing shifts in thyroid hormone economy remain so difficult to define. Evidence from animal studies suggests that chronic social defeat stress is a catalyst for both behavioral shifts and thyroid dysfunction. These outcomes are largely mediated by an impaired serotonergic system, which plays a central role in the onset of social stress-induced hypothyroidism [16].

From a cardiovascular perspective, elevated TSH levels in veterans are associated with an increased risk of hyperlipidemia and diastolic dysfunction [17]. However, clinical management remains controversial in older veterans; studies suggest that in those with chronic kidney disease (CKD), both high and low TSH levels are associated with increased mortality, creating a U-shaped risk curve [6]. Therefore, aggressive levothyroxine replacement in veterans with mild TSH elevations (4.5–10 mIU/L) requires a personalized, risk-stratified approach to avoid iatrogenic complications in this unique population.

The observed mean TSH difference of 0.47 mIU/L and effect size of Cohen’s d = 0.36 require careful interpretation. By conventional criteria, this represents a “small” effect. However, the clinical relevance of small statistical effects depends heavily on context. While a 0.47 mIU/L difference may be clinically unimportant for any individual patient, such differences at the population level can shift the proportion of individuals crossing clinical thresholds. In our data, this translated to a 7.3 percentage point absolute difference in subclinical hypothyroidism prevalence. The absolute risk difference of 7.3% yields a number needed to harm (NNH) of approximately 14, meaning that for every 14 veterans compared to controls, one additional case of subclinical hypothyroidism would be expected. In observational epidemiology of chronic conditions, odds ratios of 2–3 are commonly observed for established risk factors. The magnitude we observed (OR ≈ 2.5) is comparable to associations between smoking and various cancers or between obesity and type 2 diabetes.

The clinical significance of subclinical hypothyroidism remains debated, particularly in older adults. Current guidelines generally do not recommend treatment for subclinical hypothyroidism with TSH 10 mIU/L in patients over 65 years, as evidence of benefit is lacking and potential harms exist [18]. Some studies have linked subclinical hypothyroidism to increased cardiovascular risk, particularly in younger individuals (age 65). The association appears weaker or absent in older populations [19]. Evidence regarding cognitive decline is inconsistent. Some studies suggest associations between subclinical hypothyroidism and dementia risk, while others find no relationship [20]. Approximately 2–5% of individuals with subclinical hypothyroidism progress to overt hypothyroidism annually. Conversely, TSH normalizes spontaneously in 30–50% of cases within 2 years.

Given these uncertainties, the higher prevalence in veterans may represent a risk factor requiring monitoring rather than immediate intervention. The 12.5% prevalence in veterans (vs. population expectations of 4–10%) suggests that routine thyroid screening might identify a meaningful proportion of individuals warranting follow-up. Our control group provides a critical benchmark for interpreting the veteran findings. The control group’s mean TSH (2.06 mIU/L) and subclinical hypothyroidism prevalence (5.2%) closely match published population reference values. NHANES data for US men aged 60+ report a mean TSH of 1.9–2.3 mIU/L, and systematic reviews report subclinical hypothyroidism prevalence of 4–10% in adults over 60 years [21,22]. This concordance supports the validity of our control group as representing a typical population and suggests that the veteran group, rather than the control group, represents a departure from expected values.

Direct comparison with other veteran populations is limited by methodological heterogeneity. The few studies examining thyroid function in combat veterans have reported mixed findings. Studies of Gulf War veterans found inconsistent thyroid abnormalities, with some reporting elevated TSH and others finding no differences from controls [1,23]. Research in PTSD populations (not specifically veterans) has shown both elevated and suppressed TSH depending on study design, timing relative to trauma, and comorbidities. One study of Vietnam veterans found higher rates of autoimmune thyroiditis, suggesting a potential mechanism involving immune dysregulation [24]. The inconsistency across studies may reflect true heterogeneity in trauma effects on thyroid function, differences in population characteristics, or methodological variations. Our finding of elevated subclinical hypothyroidism prevalence adds to this literature but does not resolve the underlying mechanistic questions.

An important methodological consideration is the definition of subclinical hypothyroidism based on serum TSH concentrations. In the present study, we used the laboratory reference interval of 0.4–4.0 mIU/L, which is consistent with commonly accepted clinical reference ranges for adult populations [25]. Although this threshold is widely used in routine clinical practice, it should be acknowledged that TSH measurements are assay-dependent, and inter-method variability may influence the classification of individuals with values close to the upper reference limit. Current recommendations emphasize that TSH reference intervals should be established and validated locally for each laboratory because differences among immunoassays preclude the use of a universal reference range [26]. Furthermore, the diagnosis of subclinical hypothyroidism should not rely on a single borderline TSH measurement but should be interpreted together with free thyroid hormone concentrations, clinical findings, and, when appropriate, confirmed by repeat testing. Therefore, while the cut-off applied in our study reflects standard laboratory practice, some degree of uncertainty may exist for participants with TSH concentrations close to the upper limit of the reference interval.

Although age was included as a matching criterion rather than an individually paired variable, we additionally examined age as a potential effect modifier. Age-stratified analysis (Supplementary Table S3) showed that the odds ratio for subclinical hypothyroidism was in fact markedly higher among veterans aged 60–65 and over 65 than in younger participants, indicating that age modifies rather than explains the veteran-control association; this complements the age- and BMI-adjusted logistic regression reported above. We also considered whether the observed association could reflect an idiosyncratic subgroup or outlier effect. While a third study arm or independent validation cohort was beyond the scope and resources of this cross-sectional study, several internal robustness checks address this concern: sensitivity analyses using alternative TSH cutoffs showed consistent point estimates up to a threshold of 4.5 mIU/L (Supplementary Table S2), and the veteran-control difference was evident progressively across the upper percentiles of the TSH distribution rather than being confined to a single subgroup. We regard independent replication and validation cohorts as an important priority for future research. Regarding the possibility of an isolated outlier value, the maximum TSH value in the veterans’ group (12.87 mIU/L) corresponds to the single participant classified as overt hypothyroidism (TSH > 10 mIU/L with low FT4; Table 2); this value was verified against the original laboratory report and is not a data entry error, and no statistical outliers were excluded from the analysis. Finally, our assessment was not limited to TSH: a broad panel of clinical and biochemical measures beyond TSH and FT4 (total cholesterol, HDL and LDL cholesterol, triglycerides, AST, ALT, GGT, fasting glucose, and uric acid; Supplementary Table S1) was evaluated, of which only TSH and, to a lesser extent, FT4 showed effect sizes exceeding the threshold for a small effect.

Several additional limitations should be considered when interpreting our findings. First, information on ongoing pharmacotherapy was not systematically collected, and the use of medications known to influence thyroid function may have affected serum TSH concentrations in some participants [26]. Second, individual iodine intake was not assessed. Although Croatia has maintained an effective universal salt iodization program and is considered an iodine-sufficient country, individual variations in dietary iodine intake cannot be excluded and may have influenced thyroid function [27]. Furthermore, thyroid ultrasound examinations and measurements of thyroid autoantibodies (anti-TPO and anti-Tg) were not performed [28]. In the general Croatian adult population, thyroid autoantibodies have been reported in up to 20% of individuals, predominantly anti-thyroid peroxidase antibodies [29]. Consequently, we were unable to distinguish autoimmune thyroid disease from other causes of altered thyroid function or to identify structural thyroid abnormalities that might have influenced the observed associations. These limitations should be considered when interpreting the results and highlighting the need for future studies incorporating comprehensive clinical, laboratory, and imaging assessments.

Despite important limitations, this study has several strengths. With 672 participants, this is among the larger studies examining thyroid function in war veterans. The control group was well-matched on demographic characteristics, minimizing confounding by age and body composition. All samples were analyzed using a consistent methodology, ensuring valid between-group comparisons. Defining subclinical hypothyroidism prevalence as the main outcome of interest reduces concerns about outcome switching or selective reporting. The close match between control group values and published population norms supports the validity of our comparison. Multiple TSH cutoffs and subgroup analyses provide information about the robustness and specificity of findings.

## 5. Limitations

This study has important limitations that constrain interpretation. This was a cross-sectional study, so we cannot establish temporality or causality, and the findings may indicate association or correlation rather than a causal relationship. The observed association could reflect pre-existing differences, consequences of military service, effects of post-war experiences, or confounding by unmeasured factors. Prospective cohort studies following veterans over time could establish whether thyroid dysfunction develops after military service and whether it progresses or remits. Non-random sampling from veteran associations and healthcare facilities may not represent all Croatian war veterans. Controls from primary care may also differ from the general population. Absence of data on PTSD status, medications, socioeconomic factors, thyroid antibodies, and iodine status severely limits causal interpretation. PTSD itself, independent of veteran status, may affect thyroid function. Without a systematic psychiatric assessment, we cannot separate the effects of trauma exposure from the effects of PTSD. Without anti-TPO and anti-thyroglobulin measurements, we cannot determine whether the elevated TSH reflects autoimmune thyroiditis or other etiologies. Croatia has a history of iodine deficiency, though salt iodization programs have been implemented. Regional variations in iodine intake could contribute to thyroid dysfunction.

Smoking data were available for only 38% of participants. Smoking has complex effects on thyroid function, generally associated with lower TSH. Some biomarkers (FT3, CRP, selenium) had substantial missing data in the control group, precluding planned secondary analyses. Clinical diagnosis of subclinical hypothyroidism typically requires confirmation on repeat testing. Transient TSH elevations are common, and our prevalence estimates may overestimate true persistent subclinical hypothyroidism. Our study included only male veterans, and results may not generalize to female veterans, who may have different thyroid dysfunction patterns. We also did not assess whether the observed thyroid differences were associated with symptoms, cardiovascular outcomes, or other clinically relevant endpoints.

## 6. Conclusions

Croatian war veterans showed a higher prevalence of subclinical hypothyroidism (12.5% vs. 5.2%) and modestly elevated mean TSH compared to controls. The association persisted after adjustment for age and BMI. However, mean TSH values remained within normal limits in both groups, effect sizes were small, and the cross-sectional design with incomplete confounder data limits causal inference. These findings warrant replication in studies with a longitudinal design, comprehensive confounder assessment, and clinical outcome data. Independent, ideally multicenter, validation cohorts of veteran populations are needed to confirm the generalizability of these findings.

## Figures and Tables

**Table 1:** Participant characteristics.

Variable	Veterans (*n* = 337)	Controls (*n* = 335)	p-Value
Age (years)	62.4 ± 5.9	61.7 ± 4.2	0.078
Height (cm)	174.7 ± 7.1	176.0 ± 6.8	0.132
Weight (kg)	88.7 ± 14.2	90.1 ± 15.8	0.262
BMI (kg/m^2^)	28.7 ± 4.2	29.1 ± 4.5	0.311

Values are mean ± SD. BMI = body mass index.

**Table 2:** Distribution of thyroid status by group.

TSH Category	Veterans	%	Controls	%
Suppressed (0.4 mIU/L)	5	1.5	4	1.3
Normal (0.4–4.0 mIU/L)	288	85.7	285	93.4
Subclinical hypo (4.0–10 mIU/L)	41	12.2	16	5.2
Overt hypothyroid (10 mIU/L)	1	0.3	0	0
Missing	2	0.6	30	9.0

Percentages calculated from non-missing values. Veterans *n* = 335, Controls *n* = 305 with TSH data.

**Table 3:** Continuous thyroid parameters.

Parameter	Veterans	Controls	Diff	p	Cohen’s d	BF_10_
TSH (mIU/L)	2.53 ± 1.47	2.06 ± 1.10	0.47	0.001	0.36	2191
FT4 (units)	98.1 ± 16.9	103.6 ± 25.5	−5.5	0.002	−0.26	12.3

Values are the mean ± SD. Diff = mean difference (veterans minus controls). BF_10_ = Bayes factor favoring the alternative hypothesis. Effect sizes are small (Cohen’s d = 0.2–0.5).

## Data Availability

The authors confirm that the data supporting the findings of this study are available within the article and from the corresponding author upon request.

## Supplementary Materials

The following supporting information can be downloaded at: https://www.mdpi.com/article/10.3390/epidemiologia7040113/s1, Table S1: Other biochemical parameters; Table S2: Sensitivity analysis: Prevalence of elevated TSH at different cutoffs; Table S3: Subgroup analysis: Odds ratios for subclinical hypothyroidism by age group; Table S4: TSH percentile distributions by group; Table S5: Distributional characteristics and non-parametric tests; Figure S1: Odds ratios for subclinical hypothyroidism by age group (veterans vs. controls); Figure S2: Histograms of TSH distribution by group.
