# Supplementary material for: Higher Prevalence of Subclinical Hypothyroidism in Croatian War Veterans: A Cross-Sectional Study
Source: Epidemiologia (Basel). 2026 Aug 18;7(4):113. doi: 10.3390/epidemiologia7040113 (PMC13512066; doi:10.3390/epidemiologia7040113)
Supplement: Supplementary file 1 [file epidemiologia-07-00113-s001.zip › epidemiologia-4337892-supplementary.pdf]

Supplementary Material

Table S1. Other biochemical parameters

**Parameter**	**Veterans**	**Controls**	p	d	$\mathbf{BF}_{10}$
Glucose (mmol/L)	6.33 ± 1.5	6.17 ± 1.3	0.520	0.05	0.11
Cholesterol (mmol/L)	5.31 ± 1.1	5.29 ± 1.2	0.846	0.02	0.09
HDL (mmol/L)	1.30 ± 0.4	1.33 ± 0.4	0.478	-0.06	0.11
LDL (mmol/L)	3.29 ± 1.0	3.35 ± 1.0	0.153	-0.12	0.26
Triglycerides (mmol/L)	1.78 ± 1.0	1.86 ± 1.1	0.325	-0.08	0.15
AST (U/L)	26.9 ± 12	27.8 ± 14	0.340	-0.07	0.14
ALT (U/L)	28.3 ± 15	30.9 ± 18	0.038	-0.17	0.77
GGT (U/L)	32.9 ± 28	33.7 ± 32	0.822	-0.02	0.09
Uric acid (μmol/L)	337 ± 78	336 ± 82	0.882	0.01	0.09

Values are mean ± SD. d = Cohen's d. BF_{10} = Bayes factor. Bonferroni-corrected significance threshold: p 0.0045. No parameter reached significance after correction.

Table S2. Sensitivity analysis: Prevalence of elevated TSH at different cutoffs

**TSH Cutoff**	**n (%)**		**OR**	**95% CI**	p**-value**
(lr)2-3 **(mIU/L)**	**Veterans**	**Controls**			
3.5	61 (18.2%)	29 (9.5%)	2.12	1.32–3.40	0.002
4.0	42 (12.5%)	16 (5.2%)	2.59	1.42–4.71	0.001
4.5	30 (9.0%)	15 (4.9%)	1.90	1.00–3.61	0.062
5.0	21 (6.3%)	9 (3.0%)	2.20	0.99–4.88	0.060
10.0	1 (0.3%)	0 (0.0%)	–	–	1.000

OR = odds ratio; CI = confidence interval. Veterans n=335, Controls n=305 with TSH data. At higher cutoffs, reduced sample sizes limit statistical power. The association is robust at cutoffs ≤ 4.0 mIU/L; at higher cutoffs, point estimates remain elevated but confidence intervals cross unity.

Table S3. Subgroup analysis: Odds ratios for subclinical hypothyroidism by age group

**Age**	**n**		**Prevalence**		**OR**	**95% CI**	p
(lr)2-3 (lr)4-5 **Group**	**Vet**	**Ctrl**	**Vet**	**Ctrl**			
55 years	41	21	2.5%	4.8%	0.51	0.03–8.64	1.000
55–60 years	82	82	3.7%	8.5%	0.41	0.10–1.63	0.328
60–65 years	110	160	12.8%	1.3%	11.57	2.57–52.02	0.001
65 years	104	44	21.2%	2.4%	11.00	1.43–84.50	0.005

Subclinical hypothyroidism defined as TSH 4.0 mIU/L with FT4 within reference range (60–120). OR = odds ratio; CI = confidence interval; Vet = veterans; Ctrl = controls. Note: Wide confidence intervals in extreme age groups reflect small cell sizes. The marked heterogeneity across age groups (interaction p 0.001) should be interpreted cautiously as these analyses were not pre-specified.

Table S4. TSH percentile distributions by group

Percentile	Veterans (mIU/L)	Controls (mIU/L)	Difference (mIU/L)
5th	0.84	0.78	0.06
10th	1.07	0.96	0.11
25th	1.53	1.31	0.22
50th (median)	2.29	1.84	0.45
75th	3.09	2.44	0.65
90th	4.42	3.34	1.08
95th	5.64	4.03	1.61

The veteran-control difference increases progressively across higher percentiles, from 0.06 mIU/L at the 5th percentile to 1.61 mIU/L at the 95th percentile. This pattern suggests that the overall group difference is driven primarily by a rightward shift in the upper tail of the veteran TSH distribution rather than a uniform shift across the entire distribution.

Table S5. Distributional characteristics and non-parametric tests

**Statistic**	**Veterans**	**Controls**
Descriptive statistics for TSH (mIU/L)		
N	335	305
Mean ± SD	2.53 ± 1.47	2.06 ± 1.10
Median (IQR)	2.29 (1.53–3.09)	1.84 (1.31–2.44)
Range	0.15–12.87	0.19–7.71
Skewness	2.41	1.89
Normality tests (Shapiro-Wilk)		
W statistic	0.877	0.883
p-value	0.001	0.001
Non-parametric comparison (Mann-Whitney U)		
U statistic	61,494	
p-value	0.001	

TSH distributions were significantly right-skewed and non-normal in both groups (Shapiro-Wilk p 0.001). The Mann-Whitney U test, which makes no distributional assumptions, confirmed the significant between-group difference (p 0.001), supporting the robustness of findings to violations of normality assumptions. IQR = interquartile range.

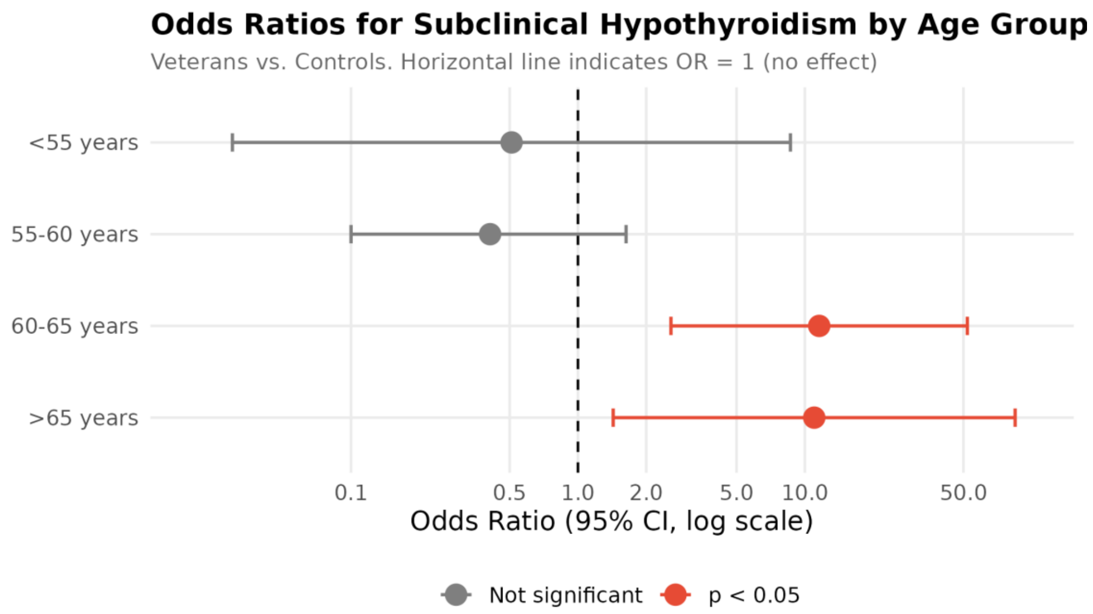

Figure S1. Odds ratios for subclinical hypothyroidism by age group (veterans vs. controls). Forest plot showing OR with 95% CI on a logarithmic scale. The vertical dashed line indicates OR = 1 (no effect). Significant associations (red) were observed only in veterans aged ≥60 years, with notably elevated ORs in the 60–65 and 65 year age groups. Wide confidence intervals reflect small cell sizes in some subgroups. These exploratory subgroup analyses were not pre-specified and should be interpreted with caution.

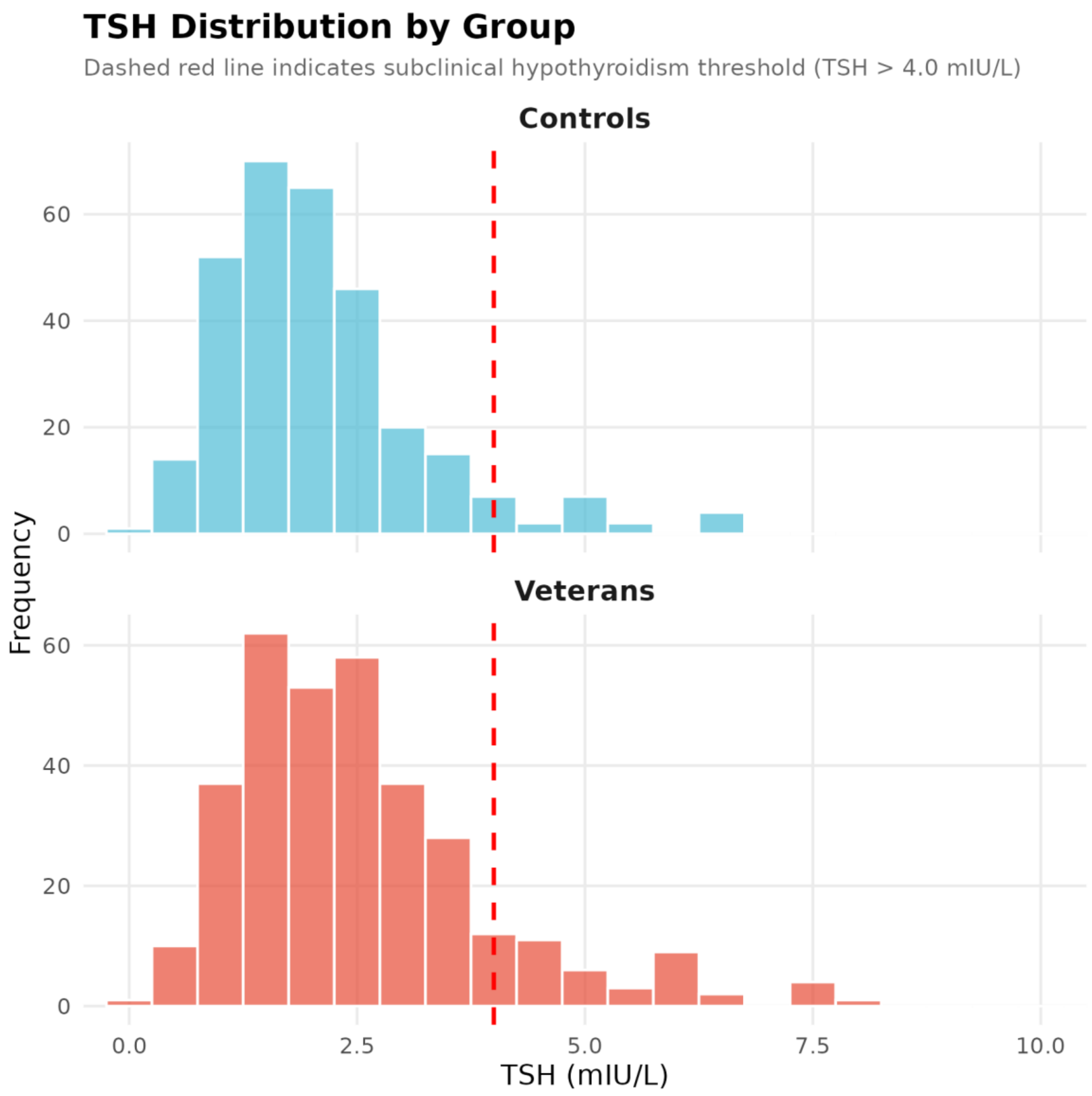

Figure S2. Histograms of TSH distribution by group. The dashed red line indicates the subclinical hypothyroidism threshold (TSH 4.0 mIU/L). Both distributions are right-skewed, but the veteran distribution shows a heavier right tail with more individuals exceeding the clinical threshold.
